# Solubility of Sulfamethazine in the Binary Mixture of Acetonitrile + Methanol from 278.15 to 318.15 K: Measurement, Dissolution Thermodynamics, Preferential Solvation, and Correlation

**DOI:** 10.3390/molecules26247588

**Published:** 2021-12-14

**Authors:** Claudia Patricia Ortiz, Rossember Edén Cardenas-Torres, Fleming Martínez, Daniel Ricardo Delgado

**Affiliations:** 1 Programa de Administración en Seguridad y Salud en el Trabajo, Grupo de Investigación en Seguridad y Salud en el Trabajo, Corporación Universitaria Minuto de Dios-UNIMINUTO, Neiva 410001, Huila, Colombia; claudia.ortiz.de@uniminuto.edu.co; 2 Grupo de Fisicoquímica y Análisis Matemático, Facultad de Ciencias y Humanidades, Fundación Universidad de América, Avenida Circunvalar No. 20-53, Bogotá 110321, Distrito Capital, Colombia; rossember.cardenas@profesores.uamerica.edu.co; 3 Grupo de Investigaciones Farmacéutico-Fisicoquímicas, Departamento de Farmacia, Facultad de Ciencias, Universidad Nacional de Colombia, Sede Bogotá, Carrera 30 No. 45-03, Bogotá 111321, Distrito Capital, Colombia; fmartinezr@unal.edu.co; 4 Programa de Ingeniería Civil, Grupo de Investigación de Ingenierías UCC-Neiva, Facultad de Ingeniería, Universidad Cooperativa de Colombia, Sede Neiva, Calle 11 No. 1-51, Neiva 410010, Huila, Colombia

**Keywords:** solubility, sulfamethazine, solution thermodynamics, preferential solvation, wilson model, NRTL model

## Abstract

Solubility of sulfamethazine (SMT) in acetonitrile (MeCN) + methanol (MeOH) cosolvents was determined at nine temperatures between 278.15 and 318.15 K. From the solubility data expressed in molar fraction, the thermodynamic functions of solution, transfer and mixing were calculated using the Gibbs and van ’t Hoff equations; on the other hand, the solubility data were modeled according to the Wilson models and NRTL. The solubility of SMT is thermo-dependent and is influenced by the solubility parameter of the cosolvent mixtures. In this case, the maximum solubility was achieved in the cosolvent mixture $w_{0.40}$ at 318.15 K and the minimum in pure MeOH at 278.15 K. According to the thermodynamic functions, the SMT solution process is endothermic in addition to being favored by the entropic factor, and as for the preferential solvation parameter, SMT tends to be preferentially solvated by MeOH in all cosolvent systems; however, $\delta x_{3,1}<0.01$, so the results are not conclusive. Finally, according to mean relative deviations (MRD%), the two models could be very useful tools for calculating the solubility of SMT in cosolvent mixtures and temperatures different from those reported in this research.

## 1. Introduction

Solubility is one of the main physicochemical parameters involved in drug development, since it is directly related to drug absorption and bioavailability. Furthermore, solubility is also an important factor in other processes such as dosage, pre-formulation, crystallization, purification, and quantification [1,2]. The importance of drug solubility has led to the development of one of the most important lines of research in the pharmaceutical industry, which consists in the development of mathematical models and has evolved towards the incursion of artificial intelligence in the development of algorithms that predict the solubility of drugs in different solvents [3,4,5,6,7,8,9].

In this context, cosolvency is relevant in solubility studies because it is a widely used strategy in the pharmaceutical industry to improve the solubility of drugs [10], in addition to being widely used in quantification processes, especially when liquid chromatography is used; therefore, solubility studies in cosolvent systems generate information that is potentially used for process optimization.

**Figure 1:**
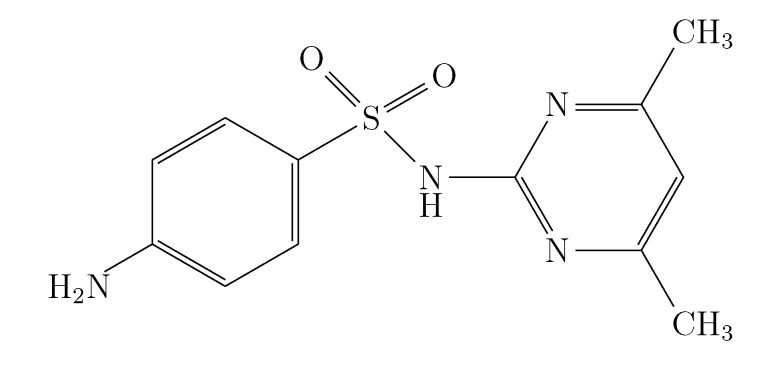
Molecular structure of the sulfamethazine.

The study drug Sulfamethazine (SMT; Figure 1) is a long-acting, broad-spectrum bacteriostatic agent used in human and animal therapy [11,12]. Its massive use, especially in veterinary medicine, has generated some problems due to its high residual power. The drug remains for a long time in the tissues of animals medicated with it, which leads to a possible impact on the end user [13,14]. Furthermore, indiscriminate use and ignorance of the dosage also leads to contamination of soils and water sources due to the presence of SMT in animal manure and urine [15,16].

In addition to the above, some processes such as quantification and analysis generate a mass of contamination due to the use of solvents. Methanol and acetonitrile are solvents that are widely used in chromatography, so data on the solubility of different bioactive substances in these solvents are very useful [17].

Therefore, in this research, the thermodynamics of the SMT solution process in acetonitrile (MeCN) + Methanol (MeOH) cosolvent mixtures at nine temperatures are analyzed in order to contribute to the strengthening of the theoretical bases on physicochemical properties of the process of solution of this drug.

## 2. Materials and Methods

### 2.1. Reagents

In this study, SMT (Sigma-Aldrich, St. Louis, MO, USA; compound 3, with purities of at least 0.990 in mass fraction), acetonitrile (Sigma-Aldrich, St. Louis, MO, USA; the solvent component 1, purity of at least 0.998 in mass fraction), and methanol (Sigma-Aldrich, USA, solvent component 2, purity of at least 0.998 in mass fraction) were used. Table 1 summarizes the sources and purities of the compounds studied.

### 2.2. Preparation of Solvent Mixtures

The MeCN (1) + MeOH (2) cosolvent mixtures are prepared 10 mL in amber colored glass flasks. A 5.00 g measure of each mixture was prepared using an analytical balance with sensitivity of ±0.1 mg (RADWAG AS 220.R2, Radom Poland). Thus, 19 cosolvent mixtures were prepared, varying the acetonitrile concentration by 0.05, from $w_{1}=0.05$ to $w_{1}=0.95$. For each concentration of acetonitrile, 3 samples were prepared.

### 2.3. Solubility Determination

The procedure was the same as that developed and published by this research group in previous research [18]. The quantification of the solubility of SMT in the MeCN + MeOH co-solvent mixtures was carried out according to the flask shaking method proposed by Higuchi and Connors [19].

- For the saturation of the cosolvent mixtures, an amount of SMT was added to each mixture until two phases were obtained (saturated solution and undissolved drug).
- Each mixture was placed inside a water recirculation bath (cryostat) (K-22/T100, Medingen, Germany) at each of the study temperatures (278.15 K, 283.15 K, 288.15 K, 293.15 K, 298.15 K, 303.15 K, 308.15 K, 313.15 K, and 318.15 K, ±0.05 K) for 72 h with constant stirring.
- After 72 h, an aliquot was taken from each vial using a syringe under semi-isothermal conditions and then filtered through a membrane with 0.45 μm pore size (Millipore Corp. Swinnex-13, St. Louis, MO, USA) to ensure the absence of undissolved solid particles. The aliquot of each solution is placed in an amber colored glass flasks and diluted gravimetrically with a 0.1 N NaOH solution to form the sodium salt of SMT, which is much more soluble than the molecular form, preventing drug precipitation.
- Finally, the concentration of each sample is determined by UV/Vis spectrophotometry (UV/Vis Spectrophotometer EMC-11-UV, Germany) according to the validated method [20].

### 2.4. Calorimetric Study

The temperature and melting enthalpies of four SMT samples were determined using differential scanning calorimetric (DSC) (DSC 204 F1 Phoenix, Berlin, Germany) (original sample, solid phase in equilibrium with saturated MeOH, solid phase in equilibrium with saturated MeCN, and solid phase in equilibrium with saturated mixture of *w*${}_{1}$ = 0.50). The samples were weighed using 5–10 mg of the drug in an aluminum crucible and placed inside the calorimeter with nitrogen current (10 mL/min). The samples were subjected to a temperature program in which they were heated from an initial temperature of 303.15 K to a temperature 480.15 K above the melting point of the analyzed drug, a heating rate of 10 K/min. The equipment was calibrated using 99.99% pure Indium.

## 3. Results and Discussion

**Figure 2:**
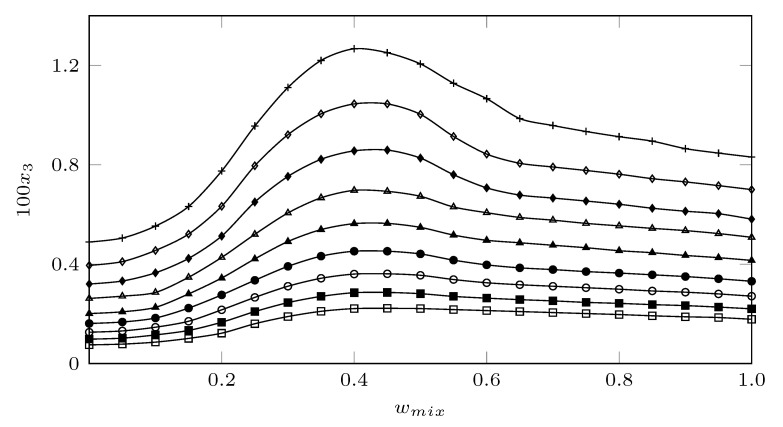
Solubility of SMT (3) expressed in mole fraction ($10^{3}x_{3}$) in MeCN (1) + MeOH (2) cosolvent mixtures as a function of mass fraction of MeCN at different temperatures. □: 278.15 K; ⯀: 283.15 K; ∘: 288.15 K; •: 293.15 K; ▲:298.15 K; △: 303.15 K; ⧫: 308.15 K; ◊: 313.15 K, and +: 318.15 K.

Figure 2 shows the experimental solubility in mole fraction of SMT in MeCN (1) + MeOH (2) cosolvent mixtures (Supporting Material; Table S1) at nine temperatures (278.15 K, 283.15 K, 288.15 K, 293.15 K, 298.15 K, 303.15 K, 308.15 K, 313.15 K and 318.15 K, ±0.05 K). The solubility data of SMT in neat methanol and neat acetonitrile were taken from the literature [21,22].

In all cases, the solubility of SMT increased with increasing temperature, indicating an endothermic process. When analyzing the behavior of the solubility as a function of the co-solvent composition, it was observed that the solubility increased with the addition of MeCN from neat MeOH to $w_{0.40}$, and from this composition to neat MeCN, MeCN behaved as an anti-solvent, since when the concentration of MeCN increased, the solubility of SMT decrease. Thus, the lowest solubility was reached in pure methanol at 278.18 K and the maximum solubility in the co-solvent mixture $w_{0.40}$ at 318.15 K. This behavior has been described for SMT in other cosolvent mixtures such as ethanol + water [23] (maximum solubility in the cosolvent mixture $w_{1}=0.80$ of ethanol in water), 1-proanol + water (maximum solubility in the cosolvent mixture $w_{1}=0.80$ of 1-proanol in water) [24], and acetonitrile + water (maximum solubility in the cosolvent mixture $w_{1}=0.90$ of acetonitrile in water) [21].

When analyzing the solubility of SMT as a function of the solubility parameter (δ), which is defined as the square root of the cohesive energy density and allows us to predict the solubility relationships, since it indicates the relative solvency power of a solcent regarding the solute/ Therefore, in these cases, SMT ($\delta_{3}$ = 27.42 MPa${}^{1/2}$) reaches its maximum solubility in co-solvent mixtures with similar polarities. That is, in EtOH + W $\delta_{mix}$ = 28.3 MPa${}^{1/2}$, in n-PrOH + W $\delta_{mix}$ = 28.3 MPa${}^{1/2}$, and in MeCN + W $\delta_{mix}$ = 26.0 MPa${}^{1/2}$. In this study, the maximum solubility of SMT was reached in a cosolvent mixture with $\delta_{mix}$ = 26.7 MPa${}^{1/2}$, which is very similar to the behavior shown in the MeCN + W system. On the other hand, in the analysis of the solubility of SMT in relation to the acidity and basicity parameters of Kamlet–Taft [25], where the alpha (α) and beta (β) parameters measure the acidity and basicity of the hydrogen bond of the solvent, respectively, SMT would behave as a Lewis base in mixtures rich in methanol due to its -NH${}_{2}$, SO${}_{2}^{-}$, and = N- groups and as a Lewis acid in mixtures rich in MeCN due to its groups -NH${}_{2}$ and >NH (Figure 1). This is due to the fact that according to the acid parameters, methanol with α = 0.990 ± 0.014 is more acidic than acetonitrile with α = 0.29 ± 0.06 [25].

**Figure 3:**
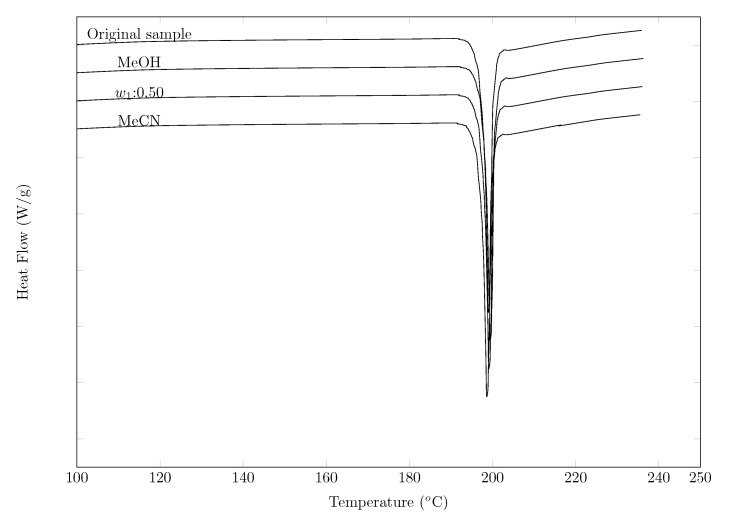
DSC thermograms of sulfamethazine (original sample, neat MeOH, $w_{1}$ = 0.50, and neat MeCN).

With the aim of verifying possible polymorphic changes, a DSC analysis was performed for the solid phase (SMT) in equilibrium with the solution saturated at three different cosolvents: pure methanol, $w_{0.50}$ and pure acetonitrile, the results were compared with the DSC of a pure SMT sample. In Figure 3, the DSC results for the four samples are shown. It can be seen that the endothermic peaks of each of the solid phase samples coincide with the pure SMT sample. When comparing the melting temperature of the four samples (solid phase of SMT in equilibrium with the saturated solution of MeOH (198.5 ${}^{{}^{\circ}}$C), $w_{0.50}$ (197.5 ${}^{{}^{\circ}}$C), MeCN (199.3 ${}^{{}^{\circ}}$C) and original sample (197.3 ${}^{{}^{\circ}}$C)), a good correlation can be observed between the data, showing that there are possibly no polymorphic changes with respect to the original sample.

When comparing the colorimetric results with those obtained by other researchers (Table 2), a good correlation is observed between the data of the present study and those of Blanco et al. [21], Delgado et al. [22], Hamada et al. [26], Sunwoo and Eisen [27], and Bustamante et al. [28]. However, when comparing the enthalpy of fusion data with those reported by Martínez and Gómez [29] and Khattab [30], a difference of 14 and 30% respectively is observed.

In relation to the melting temperature, the data reported by Maury et al. [31] and Lu and Rohani [32] are similar to those reported in this investigation.

### 3.1. Activity Coefficients

**Figure 4:**
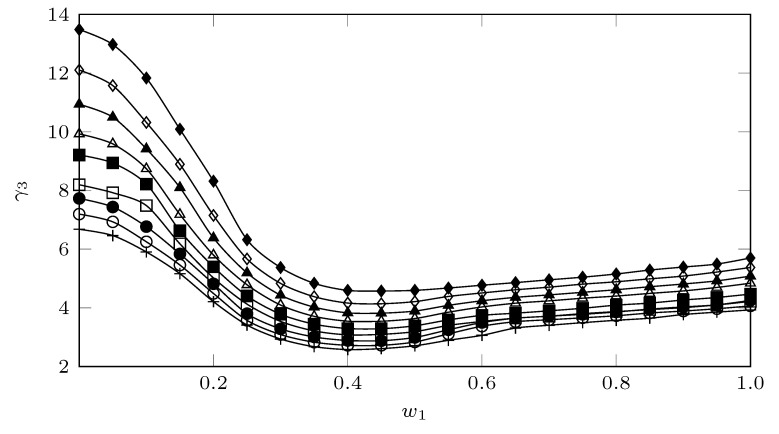
Activity coefficients of SMT (3) in MeCN (1) + MeOH (2) mixtures at several temperatures. ⧫: 278.15 K; ◊: 283.15 K; ▲: 288.15 K; △: 293.15 K; ⯀: 298.15 K; □: 303.15 K; •: 308.15 K; ∘: 313.15 K, and +: 318.15 K.

Figure 4 shows the behavior of the activity coefficients of SMT (3) in MeCN (1) + MeOH (2) cosolvent mixtures (Supporting Material; Table S2), calculated as:

(1) $$\gamma_{3}=\frac{x_{3}^{id}}{x_{3}}$$

where $x_{3}^{id}$ (ideal solubility) is calculated as

(2) $$\ln x_{3}^{id}=-\frac{\Delta_{m}H}{R}\left(\frac{T_{m}-T}{T_{m}T}\right)+\frac{\Delta Cp}{R}\left(\frac{T_{m}-T}{T}\right)-\frac{\Delta Cp}{R}\ln\frac{T_{m}}{T}$$

where $\Delta_{m}H$ (34.1 kJ/mol [21]) is the molar enthalpy of fusion of the pure solute, $T_{m}$ (471.55 K [21]) is the absolute melting point, *T* is the absolute solution temperature, *R* is the gas constant, and ΔCp is the difference between the molar heat capacity of the crystalline form and the molar heat capacity of the hypothetical super-cooled liquid form, both at the solution temperature. Since ΔCp values are not easily available in the literature, it is usual assumed that it may be approximated to the entropy of fusion, $\Delta_{m}S$, calculated as the quotient $\Delta_{m}H$/ $T_{m}$ [23].

An approximation to the analysis of the activity coefficients, from a molecular point of view, can be carried out from Equation (3) [33].

(3) $$\ln\gamma_{3}=\left(e_{11}+e_{33}-2e_{13}\right)\frac{V_{3}\phi_{1}^{2}}{RT}$$

where $e_{11}$ represents the solvent–solvent interactions, which for the present case would represent the MeCN–MeCN, MeCN–MeOH, and MeOH–MeOH interactions; $e_{33}$ represents the solute–solute interactions; $e_{13}$ represents the solute–solvent interactions (MeCN–SMT and MeOH–SMT); $V_{3}$ is the molar volume of the super-cooled liquid solute; and finally, $\phi_{1}$ is the volume fraction of the solvent. As a first approximation, for compounds with low solubility $x_{3}$, the term $V_{3}\phi_{1}^{2}/RT$ may be considered constant; thus, $\gamma_{3}$ depends mainly on $e_{11}$, $e_{33}$, and $e_{13}$.

In general terms, the solubility of SMT (3) in MeCN (1) + MeOH (2) mixtures presents relatively low activity coefficients, where the maximum value (13.48) is reached in pure MeOH at 278.15 K and the lowest (2.58) in a co-solvent mixture with $w_{0.40}$ to 318.15 where it reaches quasi-ideal values (1.0). It can be observed (Figure 4) that the temperature tends to decrease the $\gamma_{3}$ values, which according to Equation (3), would increase the interactions $e_{13}$, which would favor the solution process of the SMT. This favoring could be due to the fact that in intermediate mixtures, in addition to having a polarity similar to SMT, presenting a more favorable environment, MeCN could favor the destructuring of MeOH molecules ($e_{11}$), contributing to increasing the solute–solvent interactions ($e_{13}$).

### 3.2. Thermodynamic Functions of Solution

The thermodynamic functions of the SMT (3) solution in MeCN (1) + MeOH (2) cosolvent mixtures (Supporting Material; Table S3) are calculated according to the approach presented by Krug from the Gibbs and van ’t Hoff equations, from the experimental solubility data (Supporting Material; Table S1) [23,24].

(4) $$\Delta_{\mathrm{soln}}H^{{}^{\circ}}=-R{\left(\frac{\partial \ln x_{3}}{\partial T^{-1}-T_{hm}^{-1}}\right)}_{p}$$

(5) $$\Delta_{\mathrm{soln}}G^{{}^{\circ}}=-RT_{\mathrm{hm}}\times \mathrm{intercept}$$

(6) $$\Delta_{\mathrm{soln}}S^{{}^{\circ}}=\frac{\Delta_{\mathrm{soln}}H^{{}^{\circ}}-\Delta_{\mathrm{soln}}G^{{}^{\circ}}}{T_{\mathrm{hm}}}$$

(7) $$\zeta_{H}=\frac{|\Delta_{\mathrm{soln}}H^{{}^{\circ}}|}{|T_{\mathrm{hm}}\Delta_{\mathrm{soln}}S^{{}^{\circ}}\left|+\right|\Delta_{\mathrm{soln}}H^{{}^{\circ}}|}$$

(8) $$\zeta_{TS}=1-\zeta_{H}$$

All thermodynamic functions were calculated at the mean harmonic temperature (*T*${}_{hm}$) calculated as: $T_{hm}=n\sum_{i=1}^{n}\left(1/T\right)$, where n is the number of temperatures studied and the intercept is obtained in plots of $\ln x_{3}$ as a function of (1/*T*-1/*T*${}_{hm}$) [34]. In this research, *T*${}_{hm}$ is 297.6 K.

The solutions to thermodynamic functions are graphed and analyzed according to Perlovich’s graphical method. The relevance of Perlovich’s graphs is that in addition to being able to graph the three thermodynamic functions in a 2D graph, this allows us to identify which thermodynamic function drives the process. Thus, data in sectors I, IV, V, and VII indicate processes directed by enthalpy, and data in sectors II, III, VI, and VII indicate processes directed by entropy [35,36].

**Figure 5:**
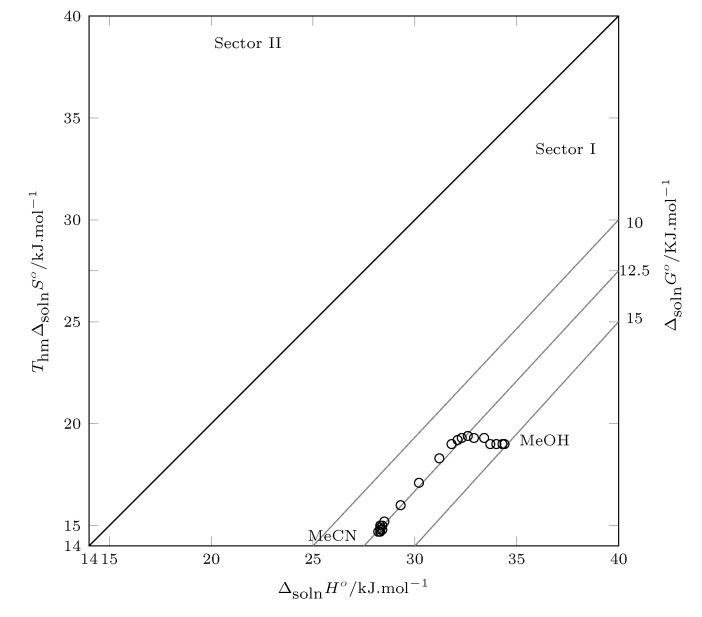
Relation between enthalpy ($\Delta_{\mathrm{soln}}H^{{}^{\circ}}$) and entropy ($T_{\mathrm{hm}}\Delta_{\mathrm{soln}}S^{{}^{\circ}}$) in terms of the process of SMT (3) solution in MeCN (1) + MeOH (2) cosolvent mixtures at 297.6 K. The isoenergetic curves for $\Delta_{\mathrm{soln}}G^{{}^{\circ}}$ are represented by dotted lines.

Therefore, Figure 5 shows the thermodynamic functions of solution. The standard Gibbs energy of solution is positive in all cases and decreases with increasing MeCN concentration in cosolvent mixtures from pure MeOH to $w_{0.40}$ and increases from this MeCN concentration to pure MeCN.

The standard enthalpy of solution is positive in all cases, indicating an endothermic process. As the concentration of MeCN in the cosolvent mixture increases, the enthalpy value decreases from pure MeOH to $w_{0.65}$; from this cosolvent mixture to pure MeCN, the enthalpy does not show significant changes (28.3–28.5 kJ/mol). The initial decrease in enthalpy may be due to an increase in solute–solvent interactions, which is consistent with the increase in the solubility of SMT; however, from $w_{0.40}$ to pure MeCN, although the solubility of SMT decreases, the enthalpy continues to decrease, so it can be inferred that solvent–solvent interactions would be favored.

Regarding the solution entropy, this thermodynamic function increases as the proportion of MeCN increases from neat MeOH to $w_{0.35}$, favoring the SMT solution process, and from this composition ($w_{0.35}$) to the neat MeCN the entropy decreases, disfavoring the solution process, which is reflected with the decrease in solubility in mixtures rich in MeCN.

In this case, all the data are in the first sector, which indicates that the overall SMT solution process is driven by enthalpy.

### 3.3. Thermodynamic Transfer Functions

From the solution thermodynamic functions, the transfer thermodynamic functions are calculated as:

(9) $$\Delta_{\mathrm{tr}}f^{{}^{\circ}}=\Delta_{\mathrm{soln}}f_{\mathrm{less}\;\mathrm{polar}}^{{}^{\circ}}-\Delta_{\mathrm{soln}}f_{\mathrm{more}\;\mathrm{polar}}^{{}^{\circ}}$$

where *f* is $\Delta_{\mathrm{soln}}G^{{}^{\circ}}$, $\Delta_{\mathrm{soln}}H^{{}^{\circ}}$ or $\Delta_{\mathrm{soln}}S^{{}^{\circ}}$.

**Figure 6:**
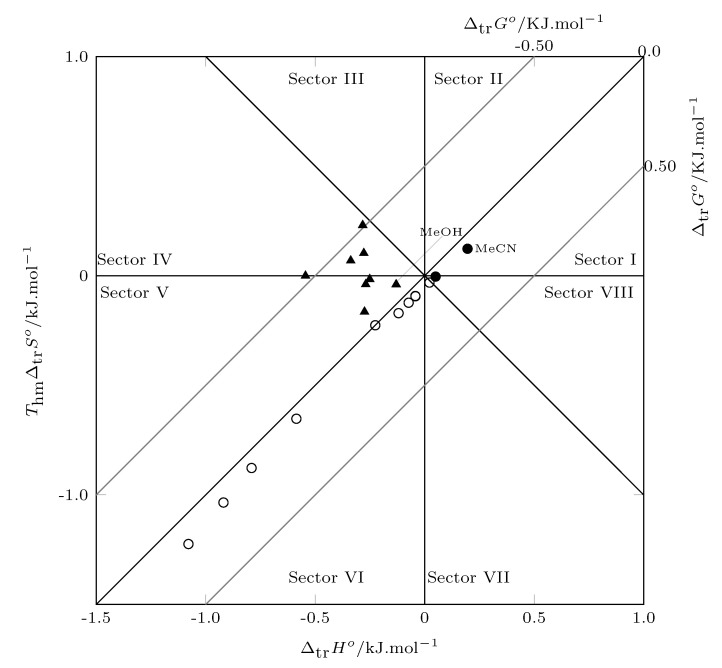
Relation between enthalpy ($\Delta_{\mathrm{tr}}H^{{}^{\circ}}$) and entropy ($T_{\mathrm{hm}}\Delta_{\mathrm{tr}}S^{{}^{\circ}}$) in terms of the process transfer of SMT (3) from more polar solvent to less polar solvent in MeCN (1) + MeOH (2) cosolvent mixtures at 297.6 K. The isoenergetic curves for $\Delta_{\mathrm{tr}}G^{{}^{\circ}}$ are represented by gray lines. (▲: $w_{0.00}\to w_{0.40}$; ∘: $w_{0.40}\to w_{0.90}$; •: $w_{0.90}\to w_{1.00}$).

Figure 6 shows the thermodynamic transfer functions (Supporting Material; Table S4), the Gibbs energy of transfer is negative from neat MeOH to $w_{0.40}$, and from this composition to neat MeCN, the Gibbs energy of transfer is positive. This indicates that SMT in mixtures rich in MeOH tends to transfer from more polar media to less polar media, and from $w_{0.40}$, SMT does not transfer to less polar media. According to the position of the data within the graph, it can be identified that the transfer of SMT from neat methanol to $w_{0.40}$ is driven by enthalpy (sector IV and V) from $w_{0.40}$ to $w_{0.90}$. The process is driven by entropy (sector VI and VII) and finally from $w_{0.90}$ to neat MeCN, and the well is driven by enthalpy (sector VIII and I).

### 3.4. Thermodynamic Mixing Functions

The hypothetical process of dissolving SMT (3) in the MeCN (1) + MeOH (2) cosolvent system can be represented as:

$$\begin{array}{c} {\mathrm{Solute}}_{\left(\mathrm{Solid}\right)}\mathrm{at}\;T{}_{hm}\to {\mathrm{Solute}}_{\left(\mathrm{Solid}\right)}\mathrm{at}\;T_{\mathrm{fus}}\to {\mathrm{Solute}}_{\left(\mathrm{Liquid}\right)}\mathrm{at}\;T_{\mathrm{fus}}\to \\ {\mathrm{Solute}}_{\left(\mathrm{Liquid}\right)}\mathrm{at}\;T{}_{hm}\to {\mathrm{Solute}}_{\left(\mathrm{Solution}\right)}\mathrm{at}\;T{}_{hm} \end{array}$$

The solution process can be expressed mathematically as:

(10) $$\Delta_{\mathrm{soln}}f^{{}^{\circ}}=f_{f}^{{}^{\circ}}+\Delta_{\mathrm{mix}}f^{{}^{\circ}}$$

where *f* is $G^{{}^{\circ}}$, $H^{{}^{\circ}}$ or $S^{{}^{\circ}}$.

**Figure 7:**
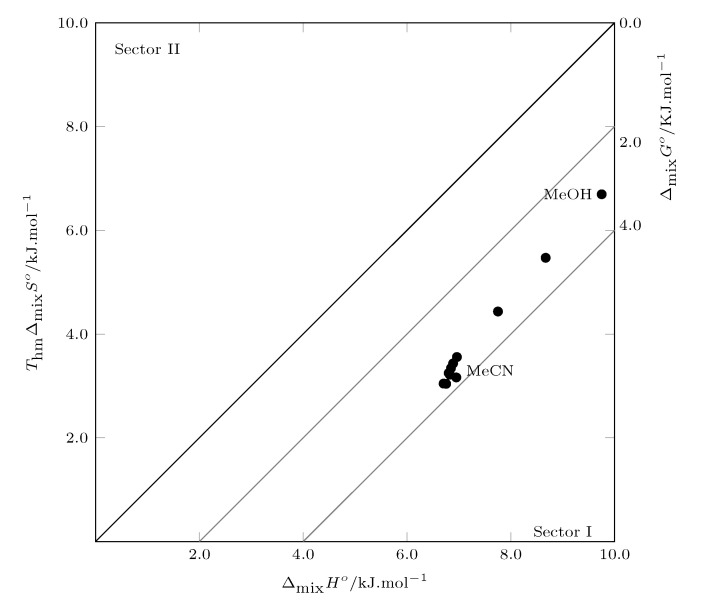
Relation between enthalpy ($\Delta_{\mathrm{mix}}H^{{}^{\circ}}$) and entropy ($T_{\mathrm{hm}}\Delta_{\mathrm{mix}}S^{{}^{\circ}}$) in terms of the process of SMT (3) solution in MeCN (1) + MeOH (2) cosolvent mixtures at 297.6 K. The isoenergetic curves for $\Delta_{\mathrm{mix}}G^{{}^{\circ}}$ are represented by gray lines.

Figure 7 shows the thermodynamic functions of mixing (Supporting Material; Table S5). It can be seen that the mixing Gibbs energy is positive in all cases, which is unfavorable to the solution process. However, the Gibbs mixing energy decreases with increasing MeCN concentration from pure MeOH to $w_{0.40}$, indicating that in this range of cosolvent mixtures, the addition of MeCN decreases the energetics related to the formation of the cavity to house the SMT molecule, which is an unfavorable endothermic process for the process of solution.

When analyzing the behavior of the mixture enthalpy, it can be observed that the enthalpy decreases with the increase in the concentration of MeCN, indicating that when MECN is added, the number of molecular interactions increases, which in mixtures rich in MeOH are possibly solute–solvents, which contrasts with the initial increase in the solubility of SMT. Finally, the solution entropy is positive in all cases, which favors the mixing process and therefore the solution process.

According to Perlovich’s analysis, the process of mixing SMT in MeCN + MeOH mixtures is driven by enthalpy, since all data are recorded in sector I [35,36]

### 3.5. Enthalpy-Entropic Compensation

By graphing $\Delta_{\mathrm{soln}}H^{{}^{\circ}}$ vs. $\Delta_{\mathrm{soln}}G^{{}^{\circ}}$, it is possible to analyze the different mechanisms involved in the cosolvent action. In addition, from this graph, the thermodynamic consequences of the molecular interactions that occur in the solution process can be analyzed, where the hydrogen bonds are the most prominent [37,38,39].

**Figure 8:**
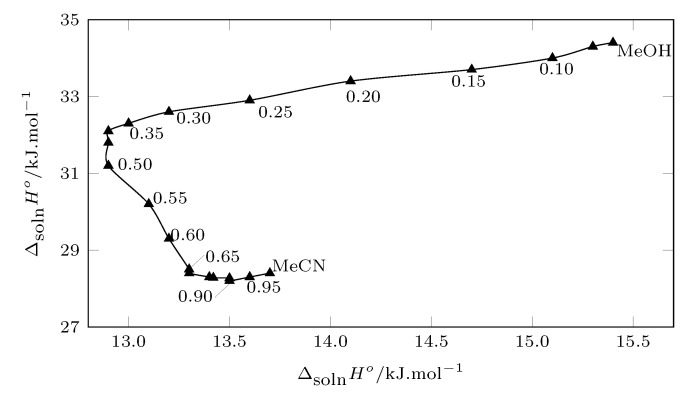
Enthalpy–entropy compensation plot for the solubility of SMT (3) in MeCN (1) + MeOH (2) mixtures at $T_{hm}$ = 297.6 K.

Figure 8 shows the enthalpy–entropy compensation of SMT (3) in MeCN (1) + MeOH (2) cosolvets mixtures, from pure MeOH to the $w_{0.40}$ cosolvent mixture. A trend with a positive slope is presented, indicating that the solution process is driven by the ethalpy. From $w_{0.40}$ to $w_{0.50}$ the Gibbs energy of the solution does not present significant variations, so a positive or negative slope cannot be clearly seen; from $w_{0.50}$ to $w_{0.75}$, a negative slope is clearly observed, indicating that the process in these mixtures it is driven by entropy; finally from $w_{0.75}$ to pure MeCN, the variation of the solution enthalpy is small, which does not allow the thermodynamic function that driven the process to be clearly identified.

It can be observed that in mixtures rich in methanol and intermediate mixtures, the enthalpy variations are much greater than those that occur in mixtures rich in MeCN, which is possibly due to the fact that when the concentration of MeCN is increased, the interactions by hydrogen bonding decreases, since MeCN is a polar aprotic solvent.

### 3.6. Preferential Solvation

From the experimental solubility data of SMT (3) in co-solvent mixtures MeCN (1) + MeOH (2), the preferential solvation of SMT by the components of the cosolvent mixture is estimated, according the inverse Kirkwood–Buff integral method (IKBI) presented by Marcus [40]. The results are expressed in terms of the preferential solvation parameter $\delta x_{1,3}$

(11) $$\delta x_{1,3}=x_{1,3}^{L}-x_{1}=-\delta x_{2,3}$$

where $x_{1}$ is the mole fraction of MeCN (1) in the bulk solvent mixture and ($x_{1,3}^{L}$) is the local mole fraction of MeCN around the solute [40,41].

If $\delta x_{1,3}>0$, then the SMT (3) is preferentially solvated by MeCN (1); on the other hand, if $\delta x_{1,3}<0$, S is preferentially solvated by MeOH (2) [42].

The mathematical equations of the IKBI model presented by Ben-Naim [43] and reformulated by Marcus are: [41]:

(12) $$\delta x_{1,3}=\frac{x_{1}x_{2}\left(G_{1,3}-G_{2,3}\right)}{x_{1}G_{1,3}+x_{2}G_{2,3}+V_{\mathrm{corr}}}$$

(13) $$G_{1,3}=RT\kappa_{T}-V_{3}+\frac{x_{2}V_{2}D}{Q}$$

(14) $$G_{2,3}=RT\kappa_{T}-V_{3}+\frac{x_{1}V_{1}D}{Q}$$

(15) $$V_{\mathrm{corr}}=2522.5{\left(r_{3}+0.1363\sqrt[{3}]{x_{1,3}^{L}V_{1}+x_{2,3}^{L}V_{2}}-0.085\right)}^{3}$$

(16) $$D={\left(\frac{\partial \Delta_{tr}G_{3,2\to 2+1}^{{}^{\circ}}}{\partial x_{1}}\right)}_{T,p}$$

(17) $$Q=RT+x_{1}x_{2}{\left(\frac{\partial^{2}G_{1,2}^{E}}{\partial x_{2}^{2}}\right)}_{T,p}$$

where $G_{1,3}$ and $G_{2,3}$ are the Kirkwood–Buff integrals (${\mathrm{cm}}^{3}{\mathrm{mol}}^{-1}$); $V_{\mathrm{corr}}$ is the volume of correlation around SMT, where the preferential solvation occurs; $\kappa_{T}$ is the isothermal compressibility of the mixtures (${\mathrm{GPa}}^{-1}$ [44]); $V_{1},V_{2}$, and $V_{3}$ are the partial molar volumes of MeCN, MeOH, and SMT, respectively; $G_{3,1\to 3,1+2}$ is the Gibbs energy of SMT transfer; and $G_{1,2}^{E}$ the molar excess Gibbs energy of their mixing (in the absence of SMT). The correlation volume is calculated by iteration because it depends on the local mole fractions present in Equations (11) and (12).

The compressibility of cosolvent mixtures can be calculated as:

(18) $$\kappa_{T}=x_{1}\kappa_{1}+x_{2}\kappa_{2}$$

$\Delta_{tr}G_{3,1\to 3,1+2}$, presents a non-linear behavior, which can be described as:

(19) $$\Delta_{tr}G_{3,1\to 3,1+2}=RT\ln\left(\frac{x_{3,1}}{x_{3,1+2}}\right)=\frac{0.020-57.09x_{1}^{2}-222.24x_{1}^{4}+268.01x_{1}^{6}}{1+5.46x_{1}^{2}+167.37x_{1}^{4}-167.52x_{1}^{6}}$$

**Figure 9:**
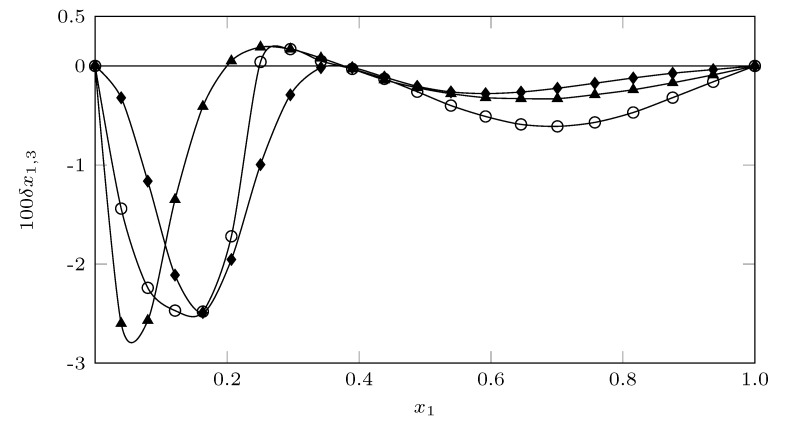
$\delta x_{1,3}$ values of SMT (3) in MeCN (1) + MeOH (2) mixtures at 298.15 K. (⧫ = SMT; ◯: SMR; ▲: SD).

Figure 9 shows the behavior of the preferential solvation parameter of SMT. According to Marcus, if $|\delta x_{1,3}|\leq 0.01$, the values are probably within the error of the determination, signifying negligible preferential solvation [40,41]; therefore, although SMT shows a tendency to be solvated in all cases by MeOH, in most cases, the values of $\delta x_{1,3}$ are less than 0.01, so it is not possible to indicate with certainty which solvent preferentially solvates the SMT, except in the mixtures from $w_{0.10}$ to $w_{0.25}$ where the values of $\delta x_{1,3}$ are greater than 0.01, indicating a solvation of the SMT by MeOH.

When comparing the preferential solvation behavior of sulfadiazine (SD [45]), sulfamerazine (SMR [46]) and sulfamethazine (SMT) in MeCN + MeOH cosolvent mixtures, the influence of the methyl groups (-CH${}_{3}$) can be seen (SD: 0 groups - CH${}_{3}$; SMR: 1 -CH${}_{3}$ groups; SMT: 2 -CH${}_{3}$ groups) in the displacement of the maximum solvation point of the three sulfonamides by MeOH, the more polar SD tends to present the peak in more polar zones, while the SMT shows the peak of maximum solvation in less polar co-solvent mixtures.

This tendency of the three sulfonamides to be preferentially solvated by MeOH in most of the mixtures, especially in SMT, which is practically solvated by MeOH in all cosolvent systems MeCN+MeOH, may be a consequence of a possible self-association of MeCN, which has been reported by Marcus [42] in different binary mixtures MeCN + organic solvents and by Hawlicka and Grabowski [47] for mixtures MeCN + MeOH. Therefore, the possible self-association of MeCN would lead to an increase in SMT-MeOH interactions, reflected in greater solvation of SMT by MeOH.

This possible self-association of MeCN could lead to a decrease in the solubility of SMR in mixtures rich in MeCN, where the MeCN–MeCN interactions would increase, disfavoring the solubility of SMT.

In Table S6 of the Supporting Material, the values of some properties associated with preferential solvation of SMT (3) in MeCN (1) + MeOH (2) mixtures at 298.15 K.

### 3.7. Modeling Correlation

The solubility of SMT in MeCN + MeOH cosolvent mixtures is quantitatively correlated using the Wilson [48,49,50] (Equation (20)) and NRTL [48,50,51] (Equation (21)) models, using Python software. The model parameters along with MRD% are tabulated in Tables S7 and S8 of the Supporting Material.

(20) $$\begin{array}{c} \ln\gamma_{3}=1-\ln(x_{3}-x_{2}\Lambda_{32}+x_{1}\Lambda_{31})-\\ \left(\frac{x_{3}}{x_{3}+x_{2}\Lambda_{32}+x_{1}\Lambda_{31}}+\frac{x_{2}\Lambda_{23}}{x_{3}\Lambda_{23}+x_{2}+x_{1}\Lambda_{21}}+\frac{x_{1}\Lambda_{33}}{x_{3}\Lambda_{13}+x_{2}\Lambda_{12}+x_{1}}\right) \end{array}$$

(21) $$\begin{array}{c} \ln\gamma_{3}=\frac{x_{2}\tau_{23}G_{23}+x_{1}\tau_{13}G_{13}}{x_{3}+x_{2}G_{23}+x_{3}+G_{13}}-x_{3}\frac{\left(x_{2}\tau_{23}G_{23}+x_{1}\tau_{13}G_{13}\right)}{{\left(x_{3}+x_{2}G_{23}+x_{3}G_{13}\right)}^{2}}\\ +\frac{x_{2}G_{32}}{x_{3}G_{32}+x_{2}+x_{1}G_{12}}\left(\tau_{32}-\frac{x_{3}\tau_{32}G_{32}+x_{1}\tau_{12}G_{12}}{x_{3}G_{32}+x_{2}+x_{1}G_{12}}\right)\\ +\frac{x_{1}G_{31}}{x_{3}G_{31}+x_{2}G_{21}+x_{1}}\left(\tau_{31}-\frac{x_{3}\tau_{31}G_{31}+x_{2}\tau_{21}G_{21}}{x_{3}G_{31}+x_{2}G_{31}+x_{1}}\right) \end{array}$$

The NRTL model presents an MRD% of 0.64, showing an excellent correlation. On the other hand, the Wilson model presents an MRD of 3.95 and an MRD% much higher than the NRTL model. However, in general terms, the MRD% of the Wilson model is low and allows them to be calculated solubility data to be obtained that are very close to the experimental solubility data.

**Figure 10:**
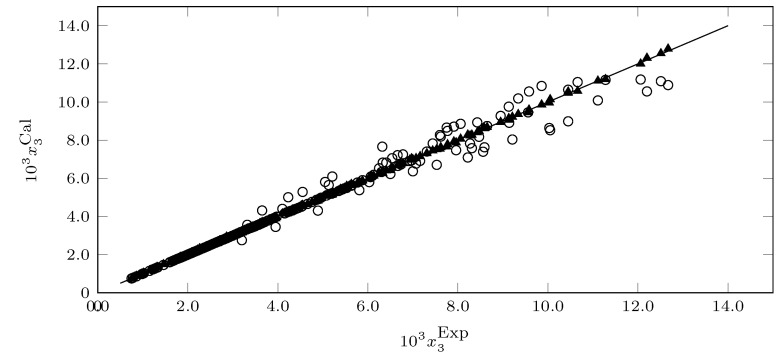
Experimental solubility data versus predicted solubility data of SMT in MeCN (1) + MeOH (2) cosolvent mixtures (∘ = Wilson model; ▲: NRTL model).

Figure 10 shows the correlation of the solubility data calculated with each of the models vs. the experimental solubility data. It can be inferred that the NRTL model presents a better correlation than the Wilson model in addition to the good correlation of the two models.

## 4. Conclusions

The SMT solution process in the MeCN (1) + MeOH (2) co-solvent mixture is thermodependent and influenced by the solubility parameter of the solvent. According to the values of the activity coefficient, the dissolution process is quasi-ideal in intermediate and MeCN-rich mixtures, where their values do not exceed the value of 6.0.

According to the thermodynamic functions of the solution, the process is endothermic and driven by the enthalpy of the solution, while the transfer thermodynamics is conditioned by the polarity of the mixture. In relation to the mixing thermodynamics, the solution process is favored by the fusion process; in addition, the mixing entropy also favors the solution process.

Regarding preferential solvation, SMT tends to be solvated by MeOH in all cases; however, in most cases, the data do not allow us to identify with certainty the solvent that preferentially solvates SMT. Finally, in relation to the correlation of the solubility of SMT, the Wilson and NRTL models allow correlating the experimental solubility of SMT in co-solvent mixtures MeCN (1) + MeOH (2) with a good degree of accuracy.

## Figures and Tables

**Table 1:** Source and purities of the compounds used in this research.

Chemical Name	CAS ${}^{a}$	Source	Purity in Mass Fraction	Analytic Technique ${}^{b}$
Sulfametazine (SMT)	57-68-1	Sigma-Aldrich, USA	>0.990	HPLC
Acetonitrile	75-05-8	Sigma-Aldrich, USA	0.998	GC
Methanol	67-56-1	Sigma-Aldrich, USA	0.998	GC

${}^{a}$ Chemical Abstracts Service Registry Number. ${}^{b}$ HPLC is high-performance liquid chromatography; GC is gas chromatography.

**Table 2:** Thermal data of sulfamethazine.

Melting Point (°C)	Enthalpy of Melting	References
198.45 ± 0.5	34.1 ± 0.5	Blanco et al. [21]
198.95 ± 0.5	33.8 ± 0.5	Blanco et al. [21]
199.65 ± 0.5	33.9 ± 0.5	Blanco et al. [21]
198.75 ± 0.5	34.1 ± 0.5	Blanco et al. [21]
196.05 ± 0.5	33.96	Delgado et al. [22]
200.00	33.96	Hamada et al. [26]
198.45	31.12	Sunwoo and Eisen et al. [27]
198.5	31.12	Bustamante et al. [28]
195.85	39.22	Martínez and Gómez [29]
195.45	44.81	Khattab [30]
197.0–198.0		Maury et al. [31]
199.1		Lu and Rohani [32]

## Data Availability

Data is contained within the article or Supplementary Material.

## Supplementary Materials

The following are available online, Table S1: Experimental solubility of SMT (3) in MeCN (1) + MeOH (2) cosolvent mixtures expressed in mole fraction (104 × 3) at different temperatures, Table S2: Coefficient activity of SMT (3) in MeCN (1) + MeOH (2) cosolvent mixtures at different temperatures, Table S3: Thermodynamic functions relative to dissolution processes of SMT (3) in MeCN (1) + MeOH (2) cosolvent mixtures at *T_hm_* = 297.6 K, Table S4: Thermodynamic functions of transfer of SMT (3) in MeCN (1) + MeOH (2) cosolvent mixtures at *T_hm_* = 297.6 K, Table S5: Thermodynamic functions relative to the mixing processes of SMT (3) in MeCN (1) + MeOH (2) cosolvent mixtures at *T_hm_* = 297.6 K, Table S6: Some properties associated with preferential solvation of SMT (3) in MeCN (1) + MeOH (2) mixtures at 298.15 K, Table S7: Parameters values of Wilson model and MRD% values for SMT in MeCN (1) + MeOH (2) mixtures at several temperatures, Table S8: Parameters values of the NRTL model and MRD% values for SMT in MeCN (1) + MeOH (2) mixtures at several temperatures.
