# Supplementary material for: Solubility of Sulfamethazine in the Binary Mixture of Acetonitrile + Methanol from 278.15 to 318.15 K: Measurement, Dissolution Thermodynamics, Preferential Solvation, and Correlation
Source: Molecules. 2021 Dec 14;26(24):7588. doi: 10.3390/molecules26247588 (PMC8706450; doi:10.3390/molecules26247588)
Supplement: Supplementary file 1 [file molecules-26-07588-s001.zip › molecules-1482866-supplementary.pdf]

Supplementary Materials

Solubility of Sulfamethazine in the Binary Mixture of Acetonitrile + Methanol from 278.15 to 318.15 K: Measurement, Dissolution Thermodynamics, Preferential Solvation,and Correlation

Claudia Patricia Ortiz [1] , Rossember Edén Cardenas-Torres [2] , Fleming Martínez [3] and Daniel Ricardo Delgado [4,*]

[1] Programa de Administración en Seguridad y Salud en el Trabajo, Grupo de Investigación en Seguridad y Salud en el Trabajo, Corporación Universitaria Minuto de Dios-UNIMINUTO, Neiva 410001, Huila, Colombia; claudia.ortiz.de@uniminuto.edu.co

[2] Grupo de Fisicoquímica y Análisis Matemático, Facultad de Ciencias y Humanidades, Fundación Universidad de América, Avenida Circunvalar No. 20-53, Bogotá 110321, Distrito Capital, Colombia; rossember.cardenas@profesores.uamerica.edu.co

[3] Grupo de Investigaciones Farmacéutico-Fisicoquímicas, Departamento de Farmacia, Facultad de Ciencias, Universidad Nacional de Colombia, Sede Bogotá, Carrera 30 No. 45-03, Bogotá 111321, Distrito Capital, Colombia; fmartinezr@unal.edu.co

[4] Programa de Ingeniería Civil, Grupo de Investigación de Ingenierías UCC-Neiva, Facultad de Ingeniería, Universidad Cooperativa de Colombia, Sede Neiva, Calle 11 No. 1-51, Neiva 410010, Huila, Colombia

* Correspondence: danielr.delgado@campusucc.edu.co; Tel.: +57-321-910-4471

Table S1

Experimental solubility of SMT (3) in MeCN (1) +MeOH (2) cosolvent mixtures expressed in mole fraction (10^4x_3) at different temperatures.

w_1[a]	Temperature/K								
	278.15	283.15	288.15	293.15	298.15	303.15	308.15	313.15	318.15
0.00	7.52±0.14	9.78±0.16	12.61±0.18	16.13±0.12	20.1±0.4	26.16±0.22	32.±0.4	39.53±0.07	48.88±0.12
0.05	7.82±0.15	10.23±0.21	13.14±0.38	16.7±0.21	20.8±0.6	27.06±0.18	33.2±0.8	41.±0.8	50.49±0.27
0.10	8.57±0.02	11.5±0.3	14.64±0.28	18.32±0.15	22.6±0.3	28.64±0.28	36.5±0.4	45.5±0.4	55.3±0.3
0.15	10.06±0.14	13.32±0.22	17.03±0.39	22.3±0.24	28.±0.4	34.69±0.13	42.3±0.8	52.1±0.8	63.2±1.1
0.20	12.2±0.23	16.56±0.33	21.61±0.33	27.62±0.27	34.37±0.33	42.72±0.16	51.3±0.3	63.3±0.2	77.5±0.2
0.25	16.05±0.3	20.9±0.5	26.57±0.94	33.5±0.5	42.1±0.9	51.98±1.18	65.±1.8	79.6±1.9	95.6±2.6
0.30	18.9±0.4	24.5±0.4	31.1±0.7	39.1±0.3	49.1±0.6	60.6±0.8	75.3±1.3	92.1±1.3	111.1±1.6
0.35	20.98±0.4	27.±0.4	34.27±0.92	43.2±0.6	53.9±0.7	66.7±1.7	82.2±1.1	100.5±1.3	122.±4.2
0.40	22.1±0.4	28.45±0.5	36.±0.8	45.2±0.4	56.3±0.8	69.7±0.6	85.6±1.8	104.5±1.7	126.7±0.9
0.45	22.2±0.4	28.6±0.6	36.1±1.	45.2±0.6	56.4±1.	69.3±1.3	85.9±1.9	104.5±2.	125.1±2.9
0.50	22.1±0.3	28.1±0.9	35.5±1.4	44.1±0.7	54.8±1.5	67.3±1.	82.7±3.3	100.3±3.1	120.6±1.5
0.55	21.7±0.4	27.±0.5	33.8±0.4	41.6±0.8	51.7±0.4	63.±0.5	76.±1.5	91.4±2.6	112.8±2.
0.60	21.26±0.29	26.3±0.4	32.52±0.28	39.7±0.5	49.57±0.08	60.7±0.8	70.7±1.	84.3±1.7	106.6±1.1
0.65	20.9±0.3	25.7±0.4	31.7±0.3	38.5±0.6	48.6±0.5	58.76±0.29	67.8±1.	80.6±1.9	98.6±0.6
0.70	20.5±0.3	25.2±0.4	31.1±0.3	37.8±0.6	47.6±0.4	57.71±0.28	66.6±1.1	79.1±1.9	95.8±0.6
0.75	20.1±0.5	24.6±0.6	30.5±0.5	37.±0.8	46.6±0.4	56.4±0.2	65.4±1.6	77.7±2.8	93.4±0.3
0.80	19.68±0.29	24.23±0.32	29.87±0.26	36.4±0.5	45.35±0.15	55.39±0.28	64.1±0.9	76.2±1.6	91.3±0.
0.85	19.18±0.26	23.7±0.6	29.2±0.4	35.7±0.9	44.58±0.25	54.4±0.3	62.5±0.8	74.4±2.	89.5±0.4
0.90	18.8±0.3	23.3±0.19	28.7±0.2	35.±0.29	43.5±0.1	53.5±0.3	61.3±1.	73.1±1.6	86.5±0.7
0.95	18.5±0.3	22.7±0.4	28.±0.3	34.1±0.6	42.6±0.2	52.3±0.3	60.3±1.1	71.6±2.1	84.7±1.
1.00	17.8±0.3	22.±0.1	27.1±0.1	33.1±0.32	41.5±0.3	50.8±0.9	58.1±0.4	70.±1.7	83.1±0.6

Ideal	0.0101	0.0118	0.0138	0.0160	0.0186	0.0214	0.0247	0.0284	0.0326

[a] w_1 is the mass fraction of MeCN (1) in the MeCN (1) + MeOH(2) mixtures free of SMT (3).

[b] is the absolute temperature, Standard uncertainties u is u(T)=0.05 K.

Table S2

Coefficient activity of SMT (3) in MeCN (1) + MeOH (2) cosolvent mixtures at different temperatures

w_1[a]	Temperature / K								
	278.15	283.15	288.15	293.15	298.15	303.15	308.15	313.15	318.15
0.00	13.48	12.10	10.94	9.93	9.21	8.19	7.73	7.19	6.68
0.05	12.97	11.57	10.50	9.59	8.94	7.92	7.44	6.93	6.46
0.10	11.83	10.32	9.42	8.74	8.22	7.48	6.77	6.25	5.90
0.15	10.09	8.89	8.10	7.18	6.63	6.18	5.84	5.46	5.16
0.20	8.32	7.15	6.38	5.80	5.40	5.02	4.82	4.49	4.21
0.25	6.32	5.67	5.19	4.78	4.40	4.12	3.80	3.57	3.41
0.30	5.37	4.83	4.43	4.09	3.78	3.54	3.28	3.09	2.94
0.35	4.83	4.38	4.02	3.71	3.44	3.21	3.01	2.83	2.67
0.40	4.59	4.16	3.83	3.54	3.29	3.08	2.89	2.72	2.58
0.45	4.57	4.14	3.82	3.54	3.29	3.10	2.88	2.72	2.61
0.50	4.60	4.21	3.89	3.63	3.39	3.19	2.99	2.84	2.70
0.55	4.67	4.39	4.08	3.85	3.59	3.40	3.25	3.11	2.89
0.60	4.77	4.51	4.24	4.03	3.74	3.53	3.50	3.37	3.06
0.65	4.86	4.61	4.36	4.16	3.82	3.65	3.65	3.53	3.31
0.70	4.95	4.70	4.44	4.24	3.90	3.72	3.71	3.59	3.41
0.75	5.04	4.81	4.53	4.33	3.98	3.80	3.78	3.66	3.49
0.80	5.15	4.89	4.62	4.40	4.09	3.87	3.86	3.73	3.57
0.85	5.29	4.99	4.72	4.49	4.16	3.94	3.96	3.82	3.65
0.90	5.39	5.08	4.81	4.58	4.27	4.01	4.03	3.89	3.77
0.95	5.49	5.22	4.92	4.69	4.35	4.10	4.10	3.97	3.85
1.00	5.70	5.37	5.08	4.84	4.47	4.22	4.25	4.06	3.93

[a] w_1 is the mass fraction of MeCN (1) in the MeCN (1) + MeOH(2) mixtures free of SMT (3).
[b] is the absolute temperature, Standard uncertainties u is u(T)=0.05 K.

Table S3

Thermodynamic functions of solution of SMT (3) in MeCN (1) + MeOH (2) cosolvent mixtures at different temperatures

w_1[a]	$\Delta_{soln}H^o$/kJ /mol	$\Delta_{soln}H^o$/kJ /mol	$\Delta_{soln}S^o$/J/ mol	$T\Delta_{soln}S^o$/k J/mol	ζ_H	ζ_{TS}
0.00	15.39±0.17	34.43±0.15	64.0±0.8	19.04±0.23	0.644	0.356
0.05	15.30±0.28	34.3±0.21	63.8±1.2	19.00±0.36	0.644	0.356
0.10	15.07±0.19	34.03±0.22	63.7±0.9	18.96±0.27	0.642	0.358
0.15	14.66±0.22	33.69±0.24	63.9±1.	19.03±0.31	0.639	0.361
0.20	14.15±0.14	33.41±0.29	64.7±0.9	19.26±0.25	0.634	0.366
0.25	13.60±0.30	32.86±0.24	64.7±1.6	19.26±0.49	0.631	0.369
0.30	13.22±0.20	32.59±0.15	65.1±1.	19.36±0.31	0.627	0.373
0.35	12.99±0.25	32.33±0.19	65.±1.3	19.35±0.39	0.626	0.374
0.40	12.88±0.19	32.06±0.15	64.5±1.	19.18±0.30	0.626	0.374
0.45	12.88±0.26	31.83±0.2	63.7±1.3	19.0±0.4	0.627	0.373
0.50	12.94±0.33	31.25±0.26	61.5±1.6	18.3±0.5	0.631	0.369
0.55	13.09±0.22	30.17±0.19	57.4±1.	17.08±0.31	0.639	0.361
0.60	13.21±0.16	29.25±0.23	53.9±0.8	16.04±0.23	0.646	0.354
0.65	13.29±0.17	28.46±0.22	51.0±0.8	15.17±0.23	0.652	0.348
0.70	13.34±0.17	28.39±0.22	50.6±0.8	15.04±0.23	0.654	0.346
0.75	13.39±0.24	28.34±0.25	50.2±1.	14.95±0.3	0.655	0.345
0.80	13.44±0.14	28.3±0.18	49.9±0.6	14.86±0.18	0.656	0.344
0.85	13.50±0.20	28.32±0.23	49.8±0.9	14.83±0.25	0.656	0.344
0.90	13.55±0.14	28.2±0.2	49.2±0.6	14.66±0.19	0.658	0.342
0.95	13.6±0.21	28.25±0.23	49.2±0.9	14.65±0.26	0.659	0.341
1.00	13.67±0.16	28.45±0.21	49.6±0.7	14.77±0.2	0.658	0.342
Ideal	9.89±0.04	21.5±0.15	39.±0.3	11.61±0.10	0.649	0.351

[a] w_1 is the mass fraction of MeCN (1) in the MeCN (1) + MeOH(2) mixtures free of SMT (3).

Table S4

Thermodynamic functions of transfer of SMT (3) in MeCN (1) + MeOH (2) cosolvent mixtures at different temperatures

More polar (w_1)→less polar (w_1)	$\Delta_{tr}H^o$/kJ/mol	$\Delta_{tr}H^o$/kJ/mol	$\Delta_{tr}S^o$/J/mol	$T\Delta_{tr}S^o$/kJ/mol
0.00→0.05	-0.09±0.33	-0.13±0.26	-0.1±1.4	0.±0.4
0.05→0.10	-0.23±0.33	-0.27±0.31	-0.1±1.5	0.±0.5
0.10→0.15	-0.41±0.29	-0.34±0.33	0.2±1.4	0.1±0.4
0.15→0.20	-0.51±0.26	-0.3±0.4	0.8±1.4	0.2±0.4
0.20→0.25	-0.5±0.4	-0.5±0.4	0.0±1.8	0.±0.5
0.25→0.30	-0.4±0.4	-0.28±0.28	0.4±1.9	0.1±0.6
0.30→0.35	-0.24±0.32	-0.25±0.25	-0.1±1.7	0.±0.5
0.35→0.40	-0.11±0.31	-0.27±0.24	-0.6±1.6	-0.2±0.5
0.40→0.45	0.0±0.32	-0.23±0.25	-0.8±1.7	-0.2±0.5
0.45→0.50	0.1±0.4	-0.59±0.32	-2.2±2.1	-0.7±0.6
0.50→0.55	0.2±0.4	-1.08±0.32	-4.1±1.9	-1.2±0.6
0.55→0.60	0.12±0.27	-0.92±0.30	-3.5±1.3	-1.0±0.4
0.60→0.65	0.09±0.23	-0.79±0.32	-3.0±1.1	-0.88±0.32
0.65→0.70	0.05±0.24	-0.07±0.31	-0.4±1.1	-0.12±0.32
0.70→0.75	0.05±0.30	-0.04±0.33	-0.3±1.3	-0.1±0.4
0.75→0.80	0.05±0.28	-0.04±0.31	-0.3±1.2	-0.09±0.35
0.80→0.85	0.05±0.25	0.02±0.29	-0.1±1.0	-0.03±0.31
0.85→0.90	0.05±0.25	-0.12±0.31	-0.6±1.1	-0.17±0.32
0.90→0.95	0.05±0.26	0.05±0.31	-0.0±1.1	0.±0.32
0.95→1.00	0.07±0.26	0.2±0.32	0.4±1.1	0.12±0.33

[a] w_1 is the mass fraction of MeCN (1) in the MeCN (1) + MeOH(2) mixtures free of SMT (3).

Table S5

Thermodynamic functions of mixing of SMT (3) in MeCN (1) + MeOH (2) cosolvent mixtures at different temperatures

w_1[a]	$\Delta_{mix}H^o$/kJ/mol	$\Delta_{mix}H^o$/kJ/mol	$\Delta_{mix}S^o$/J/mol	$T\Delta_{mix}S^o$/kJ/mol
0.00	5.5±0.17	12.93±0.21	25.0±0.8	7.43±0.25
0.05	5.41±0.28	12.8±0.26	24.8±1.3	7.39±0.38
0.10	5.18±0.19	12.53±0.27	24.7±0.9	7.35±0.28
0.15	4.77±0.22	12.19±0.28	24.9±1.1	7.42±0.33
0.20	4.26±0.15	11.91±0.33	25.7±0.9	7.65±0.27
0.25	3.72±0.33	11.37±0.29	25.7±1.7	7.6±0.5
0.30	3.34±0.2	11.09±0.22	26.1±1.1	7.75±0.32
0.35	3.1±0.25	10.84±0.25	26.±1.4	7.7±0.4
0.40	2.99±0.19	10.56±0.21	25.4±1.	7.57±0.31
0.45	2.99±0.26	10.34±0.25	24.7±1.4	7.35±0.41
0.50	3.06±0.33	9.75±0.30	22.5±1.7	6.7±0.5
0.55	3.2±0.22	8.67±0.25	18.4±1.1	5.47±0.32
0.60	3.32±0.16	7.75±0.28	14.9±0.8	4.43±0.25
0.65	3.4±0.17	6.96±0.27	12.±0.8	3.56±0.25
0.70	3.45±0.18	6.89±0.27	11.5±0.8	3.43±0.25
0.75	3.51±0.24	6.85±0.29	11.2±1.	3.34±0.31
0.80	3.56±0.14	6.8±0.24	10.9±0.7	3.25±0.20
0.85	3.61±0.21	6.82±0.28	10.8±0.9	3.22±0.27
0.90	3.66±0.15	6.71±0.25	10.2±0.7	3.05±0.21
0.95	3.71±0.21	6.76±0.28	10.2±0.9	3.04±0.27
1.00	3.79±0.16	6.95±0.26	10.6±0.7	3.16±0.22

[a] w_1 is the mass fraction of MeCN (1) in the MeCN (1) + MeOH(2) mixtures free of SMT (3).

Table S6

Some properties associated to preferential solvation of SMT (3) in MeCN (1) + MeOH (2) mixtures at 298.15 K.

x_1[a]	D kJ·mol^{-1}	$G_{1,3}$ cm^{3}·mol^{-1}	$G_{2,3}$ cm^{3}·mol^{-1}	V_{cor} cm^{3}·mol^{-1}	$\delta x_{1,3}$
0.00	0.00	-175.9	-175.9	1298.8	0.0000
0.05	-4.49	-260.8	-181.6	1342.4	-0.0032
0.10	-8.78	-357.2	-201.7	1384.6	-0.0116
0.15	-12.04	-438.5	-235.4	1426.4	-0.0211
0.20	-12.93	-463.8	-268.7	1469.1	-0.0249
0.25	-10.85	-414.7	-278.8	1513.6	-0.0195
0.30	-6.93	-322.9	-257.6	1558.8	-0.0100
0.35	-3.12	-238.3	-219.5	1603.3	-0.0029
0.40	-0.46	-184.6	-183.4	1646.8	-0.0002
0.45	0.98	-159.5	-158.4	1689.6	-0.0002
0.50	1.59	-151.7	-144.3	1732.1	-0.0011
0.55	1.72	-152.3	-138.1	1774.5	-0.0021
0.60	1.64	-156.1	-136.8	1816.9	-0.0026
0.65	1.46	-160.6	-138.4	1859.2	-0.0028
0.70	1.27	-164.8	-141.5	1901.4	-0.0026
0.75	1.09	-168.4	-145.5	1943.4	-0.0023
0.80	0.92	-171.2	-149.9	1985.3	-0.0017
0.85	0.79	-173.3	-154.2	2027.0	-0.0012
0.90	0.70	-174.7	-157.9	2068.4	-0.0007
0.95	0.70	-175.6	-159.4	2109.5	-0.0004
1.00	3.76	-176.3	-95.8	2150.2	0.0000

[a]x_1 is the mole fraction of MeCN (1) in the MeCN (1) +MeOH (2)} mixtures free of SMT (3).

Table S7

Parameters values of Willian model as well as *MRD%* values for SMT in MeCN (1) + MeOH (2) mixtures at several tempratures

T/K	τ_{12}	τ_{13}	τ_{21}	τ_{21}	τ_{31}	τ_{32}	MRD%
278.15	-3.290	-3.077	12.087	0.718	10.117	-0.721	0.84
283.15	-3.186	-2.961	11.440	0.618	9.471	-0.619	0.73
288.15	-3.090	-2.863	10.875	0.540	8.969	-0.541	0.71
293.15	-2.995	-2.763	10.348	0.492	8.476	-0.491	0.64
298.15	-2.900	-2.669	9.849	0.449	8.021	-0.446	0.66
303.15	-2.806	-2.597	9.351	0.360	7.704	-0.357	0.60
308.15	-2.712	-2.500	8.903	0.226	7.316	-0.217	0.52
313.15	-2.623	-2.415	8.493	0.303	6.976	-0.280	0.50
318.15	-2.526	-2.340	8.059	0.253	6.695	-0.210	0.55
Average							0.61

Table S8

Parameters values of NRTL model as well as *MRD%* values for SMT in MeCN (1) + MeOH (2) mixtures at several temperatures

T/K	Λ_{12}	Λ_{13}	Λ_{21}	Λ_{21}	Λ_{31}	Λ_{32}	MRD%
278.15	-0.0002	0.0006	4.53	2.247	4.59	0.414	0.08
283.15	-0.0003	0.0009	4.358	2.349	4.43	0.387	0.11
288.15	-0.0005	0.0012	4.171	2.475	4.273	0.354	0.14
293.15	-0.0007	0.0018	3.993	2.524	4.112	0.334	0.18
298.15	-0.0009	0.0024	3.825	2.518	3.962	0.323	0.18
303.15	-0.0013	0.0034	3.601	2.636	3.806	0.285	0.26
308.15	0.0529	0.5857	1.637	0.75	-0.01	-1.074	8.86
313.15	0.3195	0.1079	0.008	-1.498	1.625	0.202	8.99
318.15	0.3424	3.9649	0.019	-12.534	-1.814	1.173	8.32
Average							3.01
